# Citrullinated histone H3, a biomarker for neutrophil extracellular trap formation, predicts the risk of mortality in patients with cancer

**DOI:** 10.1111/bjh.15906

**Published:** 2019-04-09

**Authors:** Ella Grilz, Lisa‐Marie Mauracher, Florian Posch, Oliver Königsbrügge, Sabine Zöchbauer‐Müller, Christine Marosi, Irene Lang, Ingrid Pabinger, Cihan Ay

**Affiliations:** ^1^ Clinical Division of Haematology and Haemostaseology Department of Medicine I Medical University of Vienna Vienna Austria; ^2^ Division of Oncology Department of Medicine Medical University of Graz Graz Austria; ^3^ Clinical Division of Oncology Department of Medicine I Medical University of Vienna Vienna Austria; ^4^ Clinical Division of Cardiology Department of Medicine II Medical University of Vienna Vienna Austria

**Keywords:** neutrophil extracellular traps, cancer‐associated thrombosis, mortality, arterial thromboembolism, citrullinated histone H3

## Abstract

Prior studies indicate that neutrophil extracellular traps (NETs) are associated with arterial thromboembolism (ATE) and mortality. We investigated the association between NET formation biomarkers (citrullinated histone H3 [H3Cit], cell‐free DNA [cfDNA], and nucleosomes) and the risk of ATE and all‐cause mortality in patients with cancer. In this prospective cohort study, H3Cit, cfDNA and nucleosome levels were determined at study inclusion, and patients with newly diagnosed cancer or progressive disease after remission were followed for 2 years for ATE and death. Nine‐hundred and fifty‐seven patients were included. The subdistribution hazard ratios for ATE of H3Cit, cfDNA and nucleosomes were 1·0 per 100 ng/ml increase (95% confidence interval [95% CI]: 0·7–1·4, *P *= 0·949), 1·0 per 100 ng/ml (0·9–1·2, *P *= 0·494) increase and 1·1 per 1‐unit increase (1·0–1·2, *P *= 0·233), respectively. Three‐hundred and seventy‐eight (39·5%) patients died. The hazard ratio (HR) for mortality of H3Cit and cfDNA per 100 ng/ml increase was 1·1 (1·0–1·1, *P *< 0·001) and 1·1 (1·0–1·1, *P *< 0·001), respectively. The HR for mortality of nucleosome levels per 1‐unit increase was 1·0 (1·0–1·1, *P *= 0·233). H3Cit, cfDNA and nucleosome levels were not associated with the risk of ATE in patients with cancer. Elevated H3Cit and cfDNA levels were associated with higher mortality in patients with cancer.

Upon activation, neutrophils are able to generate and release decondensed chromatin fibres and granule proteins, so‐called neutrophil extracellular traps (NETs). NETs not only have the ability to kill bacteria and fungi but also interact with the blood coagulation system (Brinkmann *et al*, 2004; Martinod & Wagner, 2014). In the process of NETosis, DNA of neutrophil granulocytes is released together with various enzymes stored in granules of the neutrophil. One important enzyme, peptidylarginine deiminase 4 (PAD4) is important for a well‐described process in NETosis, the citrullination of Histone 3 (H3Cit). This process is assumed to be neutrophil‐specific and independent from other cell death mechanisms, such as apoptosis and necrosis. The release of cell free DNA (cfDNA) provides a scaffold for the adhesion of platelets and may trigger platelet activation and, therefore, activation of the blood coagulation (Fuchs *et al*, 2012). NETs have been detected in venous (VTE) and arterial thromboembolism (ATE) (Fuchs *et al*, 2010, 2012; Brill *et al*, 2012; von Brühl *et al*, 2012; Mangold *et al*, 2015; Vallés *et al*, 2017). A recent study published showed that elevated NET markers are associated with a higher risk of VTE in patients with cancer (Mauracher *et al*, 2018). Furthermore, prior studies have shown that patients with cancer have elevated levels of NET markers compared to healthy controls, and indicate that biomarkers of NET formation are associated with poor survival in patients with cancer (Yang *et al*, 2015; Richardson *et al*, 2017; Thålin *et al*, 2018). However, data on the association of biomarkers of NET formation with ATE and overall survival in patients with cancer are scarce. Therefore, in a prospective cohort study we investigated the association of biomarkers of NET formation with the risk of ATE in patients with cancer. Additionally, we investigated the association of NETs and the risk of mortality in patients with cancer. In conformity with previous studies we quantified NETs by measuring plasma levels of biomarkers reflecting NET formation, H3Cit, cfDNA and nucleosomes (Demers *et al*, 2012; Martinod *et al*, 2013; Thålin *et al*, 2016; Mauracher *et al*, 2018).

## Methods

### Study design and population

This study was conducted at the Vienna General Hospital of the Medial University of Vienna, within the framework of the Vienna Cancer and Thrombosis Study (CATS).

CATS was initiated in 2003 and is an ongoing single‐centre prospective observational cohort study, which is approved by the ethics committee (number 126/2003, ethic-kom@meduniwien.ac.at), and conducted in accordance with the Declaration of Helsinki. In brief, adult patients (aged ≥ 18 years) with a newly diagnosed cancer or a progression of disease after complete or partial remission have been included after providing written informed consent. Exclusion criteria are (i) radiotherapy or surgery within the last 2 weeks (ii) chemotherapy or a thromboembolic event within the last 3 months and (iii) an overt infection within the 6 weeks prior to study inclusion. Furthermore, all patients with an indication for long‐term anticoagulation are excluded. Patients treated with acetylsalicylic acid or other platelet inhibitors are not excluded. Temporary treatment with low molecular heparin (e.g. during hospitalization with acute medical illness) is allowed according to the study protocol. More detailed information regarding the study design and study procedures have been provided in prior publications (Ay *et al*, 2008, 2009).

In the present analysis, all patients were followed for a maximum duration of 2 years, or until the occurrence of ATE, loss of follow‐up, withdrawal of consent, or death. The primary endpoint of this analysis was death of any cause. ATE was the secondary endpoint, and was defined as a composite of myocardial infarction, ischaemic stroke, and peripheral arterial occlusion if an interventional procedure was conducted. A panel of experts in the field of cardiology, neurology and vascular medicine adjudicated all ATEs. More detailed information regarding the definition and adjudication ATE events have been reported previously (Grilz *et al*, 2018).

As CATS started with patient recruitment in 2003, plasma samples from patients recruited between 2003 and 2007 were limited, and therefore these patients were not included in this analysis. We further included only patients with complete outcome data over the 2‐year observation period. This gave a total of 976 patients that were included in CATS between 4 June 2007 and 28 October 2013. After re‐evaluation of inclusion and exclusion criteria, 19 patients were excluded from further analyses [in 17 patients no follow‐up was available; 1 patient was treated with Vitamin K antagonists and 1 patient had an implausible H3Cit level (932 times higher than the median H3Cit)].

### Laboratory analyses

At study inclusion, venous blood samples were collected via sterile venepuncture in vacuum tubes containing sodium citrate (Vacuette, Greiner BioOne, Kremsmünster, Austria). After centrifugation at 3000 × ***g*** for 10 min, platelet poor plasma was collected and stored at −80°C.

Quantification of cfDNA in plasma was performed using a Quant‐iT™ PicoGreen dsDNA Assay Kit (Thermo Fisher Scientific, Waltham, MA, USA) as described in the manufacturer's instructions. All measurements were executed in duplicates and results are expressed as ng/ml.

A cell death detection ELISAPLUS kit (Roche Diagnostics, Mannheim, Germany) was used to analyse nucleosomes in plasma samples. Nucleosomes, measured according to manufacturer's instructions, were normalised to pooled plasma obtained from 5 young male healthy controls to calculate a multiple‐of‐the‐median (MoM).

Quantification of H3Cit in plasma samples was performed as described in detail previously (Mauracher *et al*, 2018) Briefly, anti‐histone antibody from the Cell Death Detection ELISA Kit (Roche Diagnostics) was applied onto 96‐well plates (Nunc™ MicroWell™ 96‐Well Microplates; Thermo Fisher Scientific) and incubated overnight at 4°C. After blocking with incubation buffer, H3Cit standards and plasma samples were added in duplicate and incubated for 1·5 h at room temperature. After washing, plates were incubated with anti‐H3Cit antibody (1:1000, ab5103; Abcam, Cambridge, MA, USA) for 1·5 h, washed and incubated with the secondary antibody (1:5000, goat anti‐rabbit IgG horseradish peroxidase; Biorad, Hertfordshire, UK) for 1 h at room temperature. After another washing step, TMB (3,3′,5,5′‐tetramethylbenzidine; Sigma Aldrich, St. Louis, MO, USA) was applied onto the samples and incubated for 25 min, when the colourimetric reaction was stopped with 2% sulphuric acid and analysed at a wavelength of 450 nm.

The H3Cit measurement was set to 0 ng/ml when the absorbance of a patient sample was lower than the buffer blank.

### Statistical analysis

Statistical analyses were performed using spss 15 (SPSS Inc., Chicago, IL, USA) and stata 15.0 (StataCorp., College Station, TX, USA). Two‐sided *P*‐values lower than 0·05 were considered statistically significant. Count data and continuous variables were reported as absolute frequencies (percentages) and medians (25th–75th percentile), respectively. For estimation of the median follow‐up time, the reverse Kaplan–Meier method according to Schemper and Smith (1996) was used. Spearman's rank correlation coefficient was used to calculate correlations. A Kaplan–Meier estimator and uni‐ and multi‐variable Cox models were used to analyse overall survival and hazards of death, respectively. The cumulative incidence of ATE was estimated using a competing‐risk estimator with 95% confidence intervals (95% CI) (Coviello & Boggess, 2004; Ay *et al*, 2015). Gray's test was used to compare ATE incidences between groups (Gray, 1988; Ay *et al*, 2015). Subdistribution hazards of ATE were assessed using univariable and multivariable Fine & Gray competing risk regression models (Fine & Gray, 1999; Ay *et al*, 2015). Death from any cause was considered a competing event. For multivariable analyses, age, sex and metastatic disease were selected, because these variables are known for their univariable association with ATE and death (https://www.world-heart-federation.org/resources/risk-factors/; Navi *et al*, 2017; Grilz *et al*, 2018). Due to mechanistic interest, the absolute neutrophil count (ANC) was also included in all multivariable analyses. For better illustration we dichotomized H3Cit, cfDNA and nucleosomes into binary variables. Therefore, we set a cut‐off at the 75th percentile of the study population. This empiric cut‐off was also chosen in prior publications (Ay *et al*, 2010; Mauracher *et al*, 2018).

## Results

### Population

**Figure 1:**
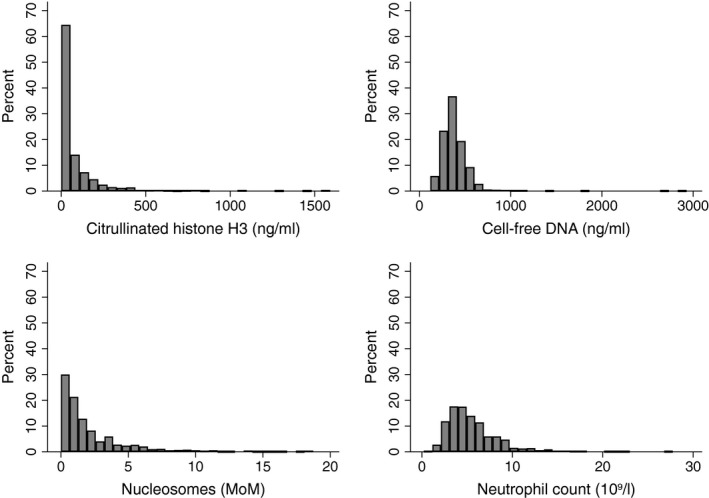
Histogram showing the distribution of the levels of citrullinated histone H3, cell‐free DNA, nucleosomes and absolute neutrophil count in patients with cancer.

Nine‐hundred and fifty‐seven patients with active cancer were included in this analysis and followed for a median of 666 days [25th–75th percentile (interquartile range, IQR): 272–731]. Detailed information about the study cohort is given in Table 1. At baseline, 706 (73·8%) patients had newly diagnosed cancer, and 251 (26·2%) had progressive disease after complete or partial remission. The median levels of H3Cit, cfDNA, nucleosomes and ANC were 25·8 ng/ml (IQR: 1·5–87·8), 359·2 ng/ml (302·2–442·6), 1·2 MoM (0·6–3·0), and 4·9 × 10^9^/l (3·6–6·8), respectively (Fig 1). H3Cit, cfDNA and nucleosomes were weakly to moderately correlated with each other. The Spearman′s correlation coefficients (rho) of H3Cit and cfDNA, H3Cit and nucleosomes, and cfDNA and nucleosomes were 0·17 (*P *< 0·001), 0·18 (*P *< 0·001) and 0·51 (*P *< 0·001), respectively. The ANC was weakly correlated with the levels of H3Cit (rho = 0·14, *P *< 0·001), cfDNA (rho = 0·18, *P *< 0·001), and nucleosomes (rho = 0·14, *P *< 0·001). H3Cit levels were highest in patients with prostate cancer (median: 44·9 ng/ml; IQR: 4·0–100·1), cfDNA levels were highest in patients with gastric cancer (419·0 ng/ml; 292·8–560·7), and nucleosome levels were highest in patients with lymphoma (1·9 MoM; 0·9–3·7). Patients with multiple myeloma had the lowest levels of H3Cit (17·7 ng/ml; 0·0–81·0) compared to other cancer types. Patients with breast cancer had the lowest levels of cfDNA (315·3 ng/ml; 259·3–371·1) and nucleosomes (0·8 MoM; 0·4–1·5). Distributions of all biomarker levels according to the type of cancer are given in Table SI.

**Table 1:** Characteristics of the total study population, patients who developed ATE and patients who died during the observation time

	All patients (*n* = 957)	ATE during follow‐up* (*n* = 22) (2·3%)	Death during follow‐up* (*n* = 378) (39·5%)
Median age at study entry, years (IQR)	61 (51–68)	66 (55–69)	63 (55–70)
Median body mass index (IQR)	25·1 (22·3–28·3)	26·4 (23·1–29·0)	24·7 (21·8–27·7)
Sex, *n* (%)			
Female	447	5 (1·1)	153 (34·2)
Male	510	17 (3·3)	225 (44·1)
Site of cancer, *n* (%)			
Lung	188	7 (3·7)	118 (62·8)
Lymphoma	164	3 (1·8)	28 (17·1)
Breast	131	0 (0·0)	20 (15·3)
Brain	126	4 (3·2)	70 (55·6)
Pancreas	76	1 (1·3)	55 (72·4)
Colon/Rectum	67	0 (0·0)	17 (25·4)
Prostate	40	1 (2·5)	8 (20·0)
Multiple myeloma	31	0 (0·0)	6 (19·4)
Stomach	27	1 (3·7)	18 (66·7)
Kidney	23	3 (13·0)	4 (17·4)
Others	84	2 (2·4)	34 (40·5)
Cancer stage (%)			
Localized	292	9 (3·1)	73 (25·0)
Distant metastasis	268	4 (1·5)	180 (67·2)
Not classifiable†	321	7 (2·2)	104 (32·4)
Unknown	76	2 (2·6)	21 (27·6)
Platelet inhibitor use at study entry, *n* (%)	158	8 (5·1)	79 (50·0)
Median H3Cit level, ng/ml‡ (IQR)	25·8 (1·5–87·8)	0·8 (0·0–29·1)	36·3 (7·1–132·9)
Median cfDNA level, ng/ml (IQR)	359·2 (302·2–442·6)	365·0 (325·3–411·8)	377·0 (317·3–460·6)
Median nucleosome level, MoM (IQR)	1·2 (0·6–3·0)	1·8 (0·8–4·3)	1·4 (0·6–3·3)
Median ANC, 10^9^/l (IQR)	4·9 (3·6–6·8)	5·4 (4·2–6·7)	5·5 (4·2–7·8)

Data on body mass index was missing in two persons. Neutrophil count is missing in 56 patients.

ANC, absolute neutrophil count; ATE, arterial thromboembolism; cfDNA, cell free DNA; H3Cit, citrullinated histone H3; IQR, interquartile range (i.e. 25th to 75th percentile), MoM, multiple of the median (i.e. nucleosome results were compared to pooled plasma from 5 young male healthy controls to obtain a multiple‐of‐the‐median).

* Percentages are related to numbers given in the first column of the same line.

† Lymphoma, brain tumours, and multiple myelomas.

‡ H3Cit level was below the detection limit in 230 patients and therefore set to zero.

### H3Cit, cfDNA, and nucleosome levels and the risk of ATE

**Figure 2:**
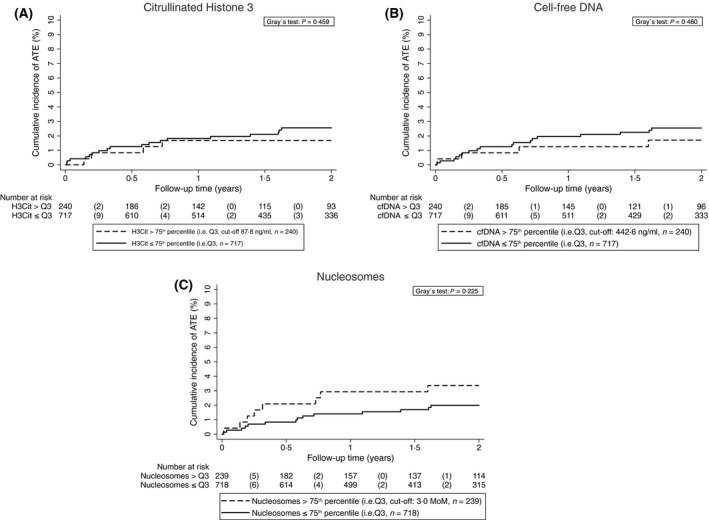
Cumulative incidence of ATE accounting for competing risk (i.e. death of any cause) according to H3Cit, cfDNA, and nucleosome levels. Note the scaling of *y*‐axis from 0% to 10% of ATE risk. ATE, arterial thromboembolism; cfDNA, cell‐free DNA; H3Cit, citrullinated histone 3; MoM, multiple of the median; Q, quartile.

Overall, 22 (2·3%) patients developed ATE during the 2‐year observation time. Nine (40·9%) patients each developed myocardial infarction and ischaemic stroke. Four (18·2%) patients developed peripheral arterial occlusion. The cumulative 6‐, 12‐ and 24‐month ATE probability was 1·2% (95% CI: 0·6–2·0), 1·8% (1·1–2·8) and 2·3% (1·5–3·5), respectively. Competing risk regression analyses were performed to analyse the association between H3Cit, cfDNA and nucleosome levels and the risk of ATE occurrence in patients with cancer. Univariable analyses found no association between the risk of ATE and H3Cit (subdistribution hazard ratio [SHR] per 100 ng/ml increase = 1·0, 95% CI: 0·7–1·4, *P *= 0·949), cfDNA (SHR per 100 ng/ml increase = 1·0, 0·9–1·2, *P *= 0·494) and nucleosomes (SHR per unit increase = 1·1, 1·0–1·2, *P *= 0·233). In patients with H3Cit levels >75th percentile the 6‐, 12‐ and 24‐month ATE risk was 0·8%, 1·7% and 1·7%, respectively. The corresponding risks for patients with H3Cit level below this cut‐off were 1·3%, 1·8% and 2·6%, respectively (SHR = 0·7, 0·2–2·0, *P *= 0·459, Fig 2A). The 6‐, 12‐ and 24‐month ATE probability was 0·8%, 1·3% and 1·7% in patients with cfDNA levels >75th percentile and 1·3%, 2·0% and 2·5% in patients below this cut‐off, respectively (SHR = 0·7, 0·2–2·0, *P *= 0·460, Fig 2B). The 6‐, 12‐ and 24‐month ATE probability was 2·1%, 2·9% and 3·4% in patients with nucleosome levels >75th percentile and 0·8%, 1·4% and 2·0% in patients below this cut‐off, respectively (SHR = 1·7, 0·7–4·1, *P *= 0·225, Fig 2C).

### H3Cit, cfDNA, and nucleosome levels and the risk of all‐cause mortality

Three‐hundred and seventy‐eight (39·5%) patients died during the observation time. The 6‐, 12‐ and 24‐month cumulative survival probabilities were 87·0% (95% CI: 84·7–89·1), 73·7% (70·7–76·4) and 57·7% (54·3–60·9), respectively. In a univariable Cox proportional hazards model, a higher H3Cit (HR per 100 ng/ml increase = 1·1, 1·0–1·1, *P *< 0·001) and a higher cfDNA (HR per 100 ng/ml increase = 1·1, 1·0–1·1, *P *< 0·001) were associated with an increased risk of mortality. A higher nucleosome level was not associated with risk of mortality (HR per unit increase = 1·0, 1·0–1·1, *P *= 0·233). After adjusting for age, sex, metastatic disease and neutrophil count the association between increasing levels of H3Cit and risk of mortality was maintained (adjusted [adj.] HR per 100 ng/ml increase = 1·1, 1·0–1·2, *P *< 0·001), but not between cfDNA and mortality (adj. HR per 100 ng/ml increase = 1·0, 1·0–1·1, *P *= 0·111). Data regarding the association of H3Cit, cfDNA and nucleosomes and the risk of mortality are presented in Table 2. In subgroup analyses, significant association between H3Cit and the risk of mortality was found in patients with lung cancer (adj. HR per 100 ng/ml increase = 1·3, 1·1–1·4, *P *< 0·001), lymphoma (adj. HR per 100 ng/ml increase = 1·5, 1·2–1·9, *P *< 0·001) and pancreatic cancer (adj. HR per 100 ng/ml increase = 1·3, 1·1–1·6, *P *< 0·001). Detailed data regarding the association of NET formation biomarkers and the risk of mortality separated by cancer type are shown in Table SI. Calculations for patients with newly diagnosed cancer and progressive disease after remission are described in Table SII.

**Table 2:** Association of H3Cit, cfDNA and nucleosome level with the risk of mortality in the total study cohort

Parameter	Univariable HR for mortality (95% CI)	*P* value	Multivariable HR for mortality (95% CI)*	*P* value
H3Cit (per 100 ng/ml increase) (*n* = 957, *n*_died_ = 378)	1·1 (1·0–1·1)	<0·001	1·1 (1·0–1·2)	<0·001
cfDNA (per 100 ng/ml increase) (*n* = 957, *n*_died_ = 378)	1·1 (1·0–1·1)	<0·001	1·0 (1·0–1·1)	0·111
Nucleosomes (per one unit increase) (*n* = 957, *n*_died_ = 378)	1·0 (1·0–1·1)	0·233	1·0 (1·0–1·1)	0·222

Calculated in univariable and multivariable Cox proportional hazard models.

cfDNA, cell free DNA; CI, confidence interval; H3Cit, citrullinated histone H3; HR, hazard ratio; *n*, number of patients; *n*_died_, number of patients who died during the observation time.

* Adjusted for age, sex, metastatic disease and neutrophil count. In multivariable analyses 56 patients are missing due to missing data of absolute neutrophil count.

**Figure 3:**
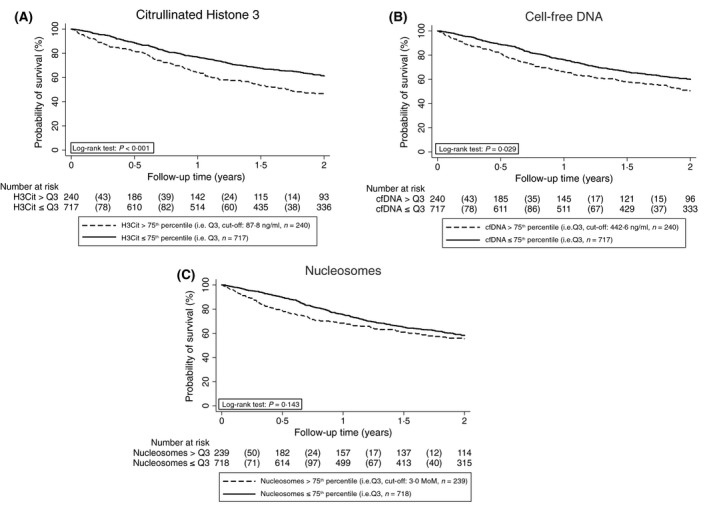
Kaplan–Meier estimates for overall survival probability of patients with cancer with H3Cit, cfDNA and nucleosome levels above and below the 75th percentile, respectively. ATE, arterial thromboembolism; cfDNA, cell‐free DNA; H3Cit, citrullinated histone 3; MoM, multiple of the median; Q, quartile.

For better illustration, we defined two groups of patients with elevated or non‐elevated NET biomarkers. Therefore, we set an empiric cut‐off at the 75th percentile of the distribution in the total study cohort. Patients with higher H3Cit baseline levels had a higher all‐cause mortality after adjusting for age, sex, metastatic disease and ANC (adj. HR = 1·6, 1·3–2·0, *P *< 0·001, Fig 3A). After 6‐, 12‐ and 24‐month, the probability of overall survival in patients with H3Cit levels >75th percentile was 81·7%, 64·4% and 46·7%, and 88·9%, 76·8% and 61·3% in those with H3Cit levels ≤75th percentile. Patients with cfDNA levels >75th percentile had an increased risk of mortality (adj. HR = 1·3, 1·0–1·6, *P *= 0·029, Fig 3B). The corresponding overall survival rates after 6‐, 12‐ and 24‐month for cfDNA levels >75th percentile were 81·7%, 66·0% and 50·5%, and 88·9%, 76·2% and 60·1% in those with cfDNA levels below the 75th percentile. No association was found between higher nucleosome levels and all‐cause mortality after adjusting for age, sex, metastatic disease and ANC (adj. HR = 1·2, 0·9–1·5, *P *= 0·143, Fig 3C). Six, 12‐ and 24‐month overall survival probabilities were 78·9%, 68·5% and 55·5% in patients with a nucleosome level >75th percentile and 89·8%, 75·4% and 58·3% in patients below this cut‐off, respectively.

## Discussion

In this prospective observational cohort study, we investigated the association of three biomarkers of NET formation (H3Cit, cfDNA and nucleosomes) with the risk of ATE and mortality in patients with cancer. While cfDNA and nucleosome release do not necessarily indicate increased NET formation, as apoptotic and necrotic cell death are other potential sources that frequently occur in patients with cancer, H3Cit is widely accepted as specific biomarker reflecting NET formation (Kerr *et al*, 1994; Schwarzenbach *et al*, 2011; Martinod *et al*, 2013; Vallés *et al*, 2017). We did not find an association between H3Cit, cfDNA and nucleosome levels and risk of ATE. However, elevated levels of H3Cit and cfDNA were associated with an increased risk of mortality in univariable analysis, while no association was found for the nucleosome levels.

Although the role of NET formation in thrombosis has been extensively investigated in mouse models, data from clinical studies are still very limited (Brill *et al*, 2012; Martinod *et al*, 2013, 2015; Meng *et al*, 2017; Hisada & Mackman, 2018). Experimental studies suggest that tumours pre‐dispose neutrophils to release NETs, which then induce a prothrombotic state (Engelmann & Massberg, 2013; Martinod & Wagner, 2014; Laridan *et al*, 2017; Pertiwi *et al*, 2018). The first evidence of a role of NETs for cancer‐associated VTE was reported recently in a study by Mauracher *et al* (2018), which found a 13% relative increase in VTE risk per 100 ng/ml increase in H3Cit level. In contrast, we did not observe an association of H3Cit with ATE in patients with cancer in our present study. We therefore conclude that biomarkers of NET formation, such as H3Cit do not play a role for prediction of ATE risk in patients with cancer. The difference between the association of biomarkers of NET formation with ATE and VTE could be explained by different pathomechanisms of ATE and VTE (Turpie & Esmon, 2011).

Previous studies in the non‐cancer setting have indicated an association of NETs with arterial thrombosis (de Boer *et al*, 2013; Borissoff *et al*, 2013; Mangold *et al*, 2015; Perez‐de‐Puig *et al*, 2015; Riegger *et al*, 2016). However, most studies investigated NETs at the site of thrombus formation (de Boer *et al*, 2013; Mangold *et al*, 2015; Perez‐de‐Puig *et al*, 2015; Riegger *et al*, 2016). One study that evaluated circulating double–stranded DNA (dsDNA), nucleosomes, citrullinated Histone 4 (H4Cit) and myeloperoxidase‐DNA complexes (MPO‐DNA complexes) as NET formation markers found that baseline levels of dsDNA, nucleosomes and MPO‐DNA are associated with the occurrence of major arterial cardiovascular events (Borissoff *et al*, 2013). We could not confirm this finding in our cohort of patients with cancer using three different assays. Furthermore, it was found that NET formation in atherosclerosis typically occurs as a consequence of low‐density lipoprotein oxidation, which is known to play a crucial role in the development and progression of atherosclerosis (Brown & Goldstein, 1986; Kita *et al*, 1987; Steinberg, 1997; Mitra *et al*, 2011; Cross & Zeki, 2016; Seo *et al*, 2018; Zhang *et al*, 2019). Patients with cancer frequently have reduced body weight or cachexia, whereby serum lipids may also decrease (Haarbo *et al*, 1989; Ng *et al*, 2007; Oda, 2018). Therefore, it could be argued that NETs play a minor role in cancer‐associated ATE due to significant changes in the stimulus of NET formation as a result of lower serum lipids.

Other reasons for the absence of an association of NET formation biomarkers with risk of ATE in patients with cancer could be differences between the pathogenesis of ATE in the general population and patients with cancer. In previous studies investigating risk factors for ATE in patients with cancer, only hypertension and a history of cardiovascular disease were significantly and independently associated with an increased risk of ATE, while body mass index, diabetes, dyslipidaemia and prior VTE did not increase ATE risk in patients with cancer (Grilz *et al*, 2018). Furthermore, anti‐cancer treatments (e.g. radiotherapy, platinum‐based chemotherapy, monoclonal antibodies, tyrosine kinase inhibitors) are known to increase the risk of cancer‐associated ATE (Scappaticci *et al*, 2007; McGale *et al*, 2011; Proverbs‐Singh *et al*, 2012; Sonpavde *et al*, 2012; Qi *et al*, 2014). As the number of arterial events was relatively low in our cohort, we cannot rule out that a large sample size could reveal the effect of biomarkers of NET formation on the development of ATE in cancer patients. Furthermore, arterial thromboembolic events are very heterogeneous, with multiple pathomechanisms. However, it was not possible to perform sub‐group analyses in certain types of arterial thrombosis due to the relatively low absolute number of arterial events. It is important to note that we did not screen for ATE, and therefore the incidence of ATE might be underestimated in our study. Patients with a high risk of cardio‐embolic stroke (e.g. patients with atrial fibrillation or artificial heart valves) are usually treated with long‐term anticoagulation. Prior studies indicate that NETs play a role in the development of cardiovascular events in patients with atrial fibrillation despite anticoagulant therapy (Arroyo *et al*, 2018). The exclusion of these patients in our study could have further reduced the number of arterial events and could thus have influenced the result regarding the association of NETs and ATE.

Next to this limitation, the strengths of our study are its prospective design and adjudication of the endpoints by an expert committee.

With regard to the prognostic impact of H3Cit and cfDNA on the risk of all‐cause mortality, our data are in line with previous findings reporting an association of biomarkers of NET formation with disease severity and reduced survival in patients with cancer (Mangold *et al*, 2015; Yang *et al*, 2015; Richardson *et al*, 2017; Thålin *et al*, 2018). Biomarkers of NET formation have been linked to tumour progression and metastatic spread, and could thereby explain poor prognosis of patients (Cools‐Lartigue *et al*, 2013; Arelaki *et al*, 2016). Cools‐Lartigue *et al* (2013) reported that NETs promote metastatic spread by sequestering cancer cells and enhancement of cancer cell adhesion to the endothelium; therefore, NETs could favour the aggressiveness of cancer (Cools‐Lartigue *et al*, 2014). These observations are supported by our study in which the association between H3Cit and mortality was high in cancer types with poor prognosis, namely lung cancer and pancreatic cancer, although we cannot exclude a multiple comparisons problem in subgroup analyses (https://seer.cancer.gov/archive/csr/1975_2014/browse_csr.php?sectionSEL=1&pageSEL=sect_01_table.05.htmlml). Furthermore, these results are substantiated by a recent study that reported higher H3Cit levels in patients with advanced cancer (Thålin *et al*, 2018).

It is important to note that direct NET quantification in clinical samples from patients has not been standardized yet. Therefore, we have indirectly quantified NETs by measuring biomarkers that have been proposed to reflect NET formation (Brinkmann *et al*, 2012; Martinod & Wagner, 2014; Vorobjeva & Pinegin, 2014). We used an H3Cit ELISA that has been employed in previous clinical studies (Thålin *et al*, 2016, 2017; Paues Göranson *et al*, 2018). As no biological cut‐offs for elevated levels of biomarkers for NET formation have been established, we chose an empirical cut‐off at the 75th percentile of the distribution of H3Cit, cfDNA and nucleosome levels in the study population to define elevated levels of these biomarkers. To minimize the potential for type 1 or type 2 errors, we also analysed the biomarkers as continuous variables in the main analysis, and regression coefficients from these analyses can be interpreted without potential bias from cut‐offs. A potential limitation is that we measured the biomarkers of NET formation only at one single time point, before initiation of anti‐tumour treatment. Thus, we cannot exclude potential changes of H3Cit, cfDNA and nucleosome levels during cancer evolution and therapy, and the resulting impact on study outcomes. Finally, a major limitation is that we did not prospectively collect data on performance status or response to cancer treatments. Therefore, we cannot address the question of whether the adverse prognostic impact of higher levels of biomarkers of NET formation on mortality is mediated by poor performance status or correlates with poor response to anti‐cancer therapy. By excluding patients with an overt bacterial or viral infection, we aimed at reducing the influence of infection on biomarkers measured in CATS, including those of NETs formation. Nonetheless, our study design did not enable us to reveal the impact of infection or of inflammation during the course of disease or other acute conditions on NET formation during the course of disease and to assess the resulting effect on study outcomes.

In summary, we found a univariable association between two biomarkers of NET formation, H3Cit and cfDNA, and the risk of mortality in patients with cancer. However, in multivariable analyses only H3Cit emerged as independently associated biomarker for mortality risk in patients with cancer. Our study adds further evidence that NETs are associated with poor survival in patients with cancer; nevertheless future research is needed to validate our conclusions and to further elucidate the role of NETs in patients with cancer.

## Disclosures

The authors have no conflict of interest to declare.

## Authorship contributions

E.G. acquired, analysed and interpreted data, performed statistical analyses, coordinated the study, performed the experiments and drafted the manuscript; L.‐M.M. designed and performed the experiments; F.P. performed statistical analyses and critically revised the manuscript; O.K. acquired and interpreted data, and recruited patients; S.Z.‐M. critically revised the manuscript for important intellectual content, and contributed to the study design; C.M. critically revised the manuscript for important intellectual content, and contributed to the study design; I.M.L. critically revised the manuscript for important intellectual content, was a member of the adjudication committee, and contributed to the study design; I.P. designed and conceived the study, obtained funding, critically revised the manuscript for important intellectual content, and provided administrative support. C.A. acquired data, designed and supervised the study, obtained funding, critically revised the manuscript for important intellectual content, and provided administrative support; all authors reviewed and edited the manuscript and approved the final article.

## Data access and responsibility

Ella Grilz and Cihan Ay had full access to all the data in the study and take the responsibility for the integrity of the data and the accuracy of the data analysis.

## Supporting information


**Table SI.** Association of H3Cit, cfDNA, and nucleosome level and the risk of mortality in the total study cohort and separated by cancer type.
**Table SII.** Association of NET formation parameters with the risk of mortality and ATE – in patients with newly diagnosed cancer (*n* = 706) *versus* patients with progression of disease after complete or partial remission (*n* = 251).
