# Supplementary material for: Citrullinated histone H3, a biomarker for neutrophil extracellular trap formation, predicts the risk of mortality in patients with cancer
Source: Br J Haematol. 2019 Apr 9;186(2):311–20. doi: 10.1111/bjh.15906 (PMC6618331; doi:10.1111/bjh.15906)
Supplement: Supplementary file 1 — Table SI. Association of H3Cit, cfDNA, and nucleosome level and the risk of mortality in the total study cohort and separated by cancer type. Table SII. Association of NET formation parameters with the risk of mortality and ATE – in patients with newly diagnosed cancer (n = 706) versus patients with progression of disease after complete or partial remission (n = 251). [file BJH-186-311-s001.docx]

**Supplementary Material**

**Supplementary table 1**

**Association of H3Cit, cfDNA, and nucleosome level and the risk of mortality in the total study cohort and separated by cancer type.**

Calculated in univariable and multivariable Cox proportional hazard models.

^a^ Adjusted for age, sex, metastatic disease, and neutrophil count. In multivariable analyses 56 patients are missing due to missing data of absolute neutrophil count.

^b^ Results regarding H3Cit and cfDNA were calculated per 100ng/mL increase; results regarding nucleosome levels are calculated per one unit increase

^c^Analyses of brain cancer were adjusted for age, sex, and neutrophil count; analyses of breast and prostate cancer were adjusted for age, metastatic disease, and neutrophil count.

Abbreviations: HR = hazard ratio, CI = confidence interval, MoM=multiple of the mean, n = number of patients, n_died_ = number of patients who died during the observation time.

**Supplementary table 1**

| **Parameter** | **Median (IQR) of the levels of citrullinated histone H3 [ng/mL], cell-free DNA [ng/mL], and nucleosomes [MoM]** | **Univariable HR for mortality (95% CI)^b^** | ***p* value** | **Multivariable HR for mortality (95% CI)^a,b^** | ***p* value** |
| --- | --- | --- | --- | --- | --- |
| H3Cit of the total study population (n=957, n_died_=378) | 25.8 (1.5-87.8) | 1.1 (1.0-1.1) | <.001 | 1.1 (1.0-1.2) | <.001 |
| Lung cancer (n=188) | 22.5 (0.0-77.2) | 1.3 (1.1-1.4) | <.001 | 1.3 (1.1-1.4) | <.001 |
| Lymphoma (n=164) | 21.0 (0.0-61.8) | 1.1 (0.9-1.3) | .282 | 1.5 (1.2-1.9) | <.001 |
| Breast cancer (n=131) | 23.7 (4.3-88.8) | 1.0 (0.9-1.3) | .667 | 1.2 (1.0-1.5)^c^ | .101 |
| Brain cancer (n=126) | 38.3 (8.2-109.7) | 1.1 (1.0-1.3) | .099 | 1.1 (0.9-1.2)^c^ | .463 |
| Pancreatic cancer (n=76) | 32.4 (9.0-131.1) | 1.4 (1.2-1.6) | <.001 | 1.3 (1.1-1.6) | <.001 |
| Colon/Rectum cancer (n=67) | 21.9 (0.6-83.8) | 1.0 (0.8-1.4) | .742 | 1.0 (0.7-1.4) | .939 |
| Prostate cancer (n=40) | 44.9 (4.0-100.1) | 1.2 (0.7-2.0) | .525 | 0.9 (0.4-1.8)^c^ | .758 |
| Multiple Myeloma (n=31) | 17.2 (1.9-43.7) | 2.3 (0.8-6.8) | .124 | 1.4 (0.5-4.0) | .533 |
| Stomach cancer (n=27) | 30.4 (0.0-96.3) | 1.0 (1.0-1.2) | .986 | 0.9 (0.8-1.1) | .416 |
| Kidney cancer (n=23) | 17.7 (0.0-81.0) | 0.3 (0.0-6.4) | .432 | 0.1 (0.0-17.2) | .344 |
| Others (n=84) | 41.5 (10.7-146.3) | 1.1 (0.9-1.3) | .278 | 1.0 (0.8-1.2) | .968 |
| cfDNA of the total study population (n=957, n_died_=378) | 359.2 (302.2-442.6) | 1.1 (1.0-1.1) | <.001 | 1.0 (1.0-1.1) | .111 |
| Lung cancer (n=188) | 378.2 (319.8-462.9) | 1.1 (1.0-1.3) | .063 | 1.1 (0.9-1.2) | .285 |
| Lymphoma (n=164) | 378.4 (310.9-460.2) | 1.0 (0.8-1.2) | .980 | 1.1 (0.8-1.4) | .569 |
| Breast cancer (n=131) | 315.3 (259.3-371.1) | 1.3 (1.0-1.6) | .037 | 1.3 (1.0-1.7)^c^ | .061 |
| Brain cancer (n=126) | 369.3 (317.9-438.2) | 1.3 (1.1-1.6) | .005 | 1.2 (1.0-1.5)^c^ | .055 |
| Pancreatic cancer (n=76) | 405.2 (316.7-475.1) | 1.2 (0.9-1.5) | .139 | 1.1 (0.8-1.4) | .686 |
| Colon/Rectum cancer (n=67) | 357.2 (310.3-460.6) | 1.0 (1.0-1.1) | .288 | 1.0 (0.9-1.1) | .996 |
| Prostate cancer (n=40) | 328.2 (297.5-393.6) | 0.8 (0.4-1.8) | .603 | 0.6 (0.2-1.7)^c^ | .346 |
| Multiple Myeloma (n=31) | 344.5 (265.4-439.2) | 1.0 (0.6-1.5) | .937 | 0.9 (0.5-1.5) | .670 |
| Stomach cancer (n=27) | 419.0 (292.8-560.7) | 1.4 (1.0-1.8) | .024 | 1.5 (1.1-2.0) | .013 |
| Kidney cancer (n=23) | 388.6 (313.3-442.3) | 0.5 (0.1-1.7) | .232 | 1.1 (0.8-1.4) | .686 |
| Others (n=84) | 340.2 (290.8-405.2) | 1.1 (0.9-1.5) | .374 | 1.1 (0.8-1.5) | .501 |
| Nucleosomes of the total study population (n=957, n_died_=378) | 1.2 (0.6-3.0) | 1.0 (1.0-1.1) | .233 | 1.0 (1.0-1.1) | .222 |
| Lung cancer (n=188) | 1.3 (0.6-3.2) | 1.0 (1.0-1.1) | .203 | 1.0 (1.0-1.1) | .615 |
| Lymphoma (n=164) | 1.9 (0.9-3.7) | 1.0 (0.9-1.1) | .545 | 1.1 (1.0-1.2) | .261 |
| Breast cancer (n=131) | 0.8 (0.4-1.5) | 1.1 (1.0-1.2) | .074 | 1.1 (1.0-1.2)^c^ | .159 |
| Brain cancer (n=126) | 1.4 (0.7-2.9) | 1.1 (1.0-1.2) | .112 | 1.1 (1.0-1.2)^c^ | .269 |
| Pancreatic cancer (n=76) | 1.0 (0.5-2.9) | 1.1 (1.0-1.2) | .047 | 1.2 (1.0-1.3) | .007 |
| Colon/Rectum cancer (n=67) | 0.8 (0.5-2.4) | 1.0 (0.8-1.2) | .955 | 0.9 (0.7-1.1) | .423 |
| Prostate cancer (n=40) | 1.3 (0.7-3.6) | 0.8 (0.6-1.2) | .382 | 0.5 (0.2-1.0)^c^ | .050 |
| Multiple Myeloma (n=31) | 1.1 (0.4-2.3) | 1.0 (0.7-1.5) | .925 | 0.9 (0.6-1.5) | .800 |
| Stomach cancer (n=27) | 1.8 (0.7-3.8) | 1.0 (0.9-1.2) | .915 | 1.1 (0.9-1.3) | .370 |
| Kidney cancer (n=23) | 1.9 (0.9-3.1) | 0.7 (0.4-1.5) | .398 | 1.3 (0.4-4.3) | .636 |
| Others (n=84) | 0.9 (0.4-2.2) | 1.0 (0.8-1.1) | .599 | 0.9 (0.8-1.1) | .444 |

**Supplementary table 2**

**Association of NET formation parameters with the risk of mortality and ATE - in patients with newly diagnosed cancer (n=706) versus patients with progression of disease after complete or partial remission (n=251).**

The median levels of H3Cit were 26.2 ng/mL (IQR: 1.9-88.3) in patients with newly diagnosed cancer and 24.1 ng/mL (1.1-84.8) in patients with progressive disease; Wilcoxon Rank-Sum test: p=.611. The median levels of cfDNA were 363.5 ng/mL (301.6-445.8) in patients with newly diagnosed cancer and 354.5 ng/mL (303.6-438.6) in patients with progressive disease; Wilcoxon Rank-Sum test: p=.843. The median levels of nucleosomes were 1.1 MoM (0.5-2.6) in patients with newly diagnosed cancer and 1.5 MoM (0.7-3.6) in patients with progressive disease; Wilcoxon Rank-Sum test: p=.004.

Hazard ratios are calculated in univariable and multivariable Cox proportional hazard models.

^a^ Adjusted for age, sex, metastatic disease, and neutrophil count. In multivariable analyses 56 patients are missing due to missing data of absolute neutrophil count.

^b^ Results regarding H3Cit and cfDNA were calculated per 100ng/mL increase; results regarding nucleosome levels are calculated per one unit increase

Abbreviations: HR = hazard ratio, CI = confidence interval

| **Parameter** | **Univariable HR for mortality in patients with newly diagnosed cancer (95% CI)^b^** | ***p***  **value** | **Multivariable HR for mortality in patients with newly diagnosed cancer (95% CI)^a,b^** | ***p***  **value** | **Univariable HR for mortality in patients with progressive disease (95% CI)^b^** | ***p***  **value** | **Multivariable HR for mortality in patients with progressive disease (95% CI)^a,b^** | ***p***  **value** |
| --- | --- | --- | --- | --- | --- | --- | --- | --- |
| H3Cit | 1.1 (1.0-1.2) | .001 | 1.1 (1.0-1.2) | .005 | 1.1 (1.0-1.2) | .145 | 1.1 (1.0-1.3) | .032 |
| cfDNA | 1.1 (1.0-1.1) | <.001 | 1.0 (1.0-1.1) | .218 | 1.0 (1.0-1.2) | .614 | 1.1 (1.0-1.3) | .186 |
| Nucleosomes | 1.1 (1.0-1.1) | .027 | 1.0 (1.0-1.1) | .260 | 1.0 (1.0-1.1) | .561 | 1.0 (1.0-1.1) | .357 |
